# Exploring Acoustic Correlates of Depression and Preliminary Screening Models Using XGBoost and SHAP

**DOI:** 10.3390/bs15121648

**Published:** 2025-11-30

**Authors:** Kwang-Ho Seok, Jaeeun Shin, Sung-Man Bae

**Affiliations:** 1 School of AI Convergence, Global Cyber University, Cheonan 31228, Republic of Korea; mono-skh@gw.global.ac.kr; 2 Department of Psychology, Chung-Ang University, Seoul 06974, Republic of Korea; rheai@cau.ac.kr; 3 Department of Psychology and Psychotherapy, College of Health Science, Dankook University, Cheonan 31116, Republic of Korea

**Keywords:** depression prediction, voice analysis, machine learning, SHAP, acoustic features

## Abstract

This exploratory study investigated whether voice-derived acoustic features reflect depressive symptom severity and whether they carry preliminary predictive signal for distinguishing individuals with Major Depressive Disorder (MDD) from healthy controls (HC). Using the publicly available MODMA dataset (23 MDD; 29 HC), 6553 acoustic features were extracted with openSMILE. Spearman correlation and group-difference analyses identified several MFCC-derived spectral features as moderately and systematically associated with PHQ-9 scores, indicating their potential relevance as severity-linked acoustic markers. To complement these findings, a supplementary severity-based classification using a PHQ-9 ≥ 10 threshold showed that a logistic regression model trained on the top five correlated MFCC features achieved a cross-validated AUC of 0.78 (SD = 0.15), supporting their association with clinically defined symptom burden. Four machine learning pipelines were further evaluated for an exploratory MDD–HC classification task. Among them, the PCA + XGBoost model demonstrated the most stable generalization (test AUC = 0.60), although predictive performance remained limited within the constraints of the small and high-dimensional dataset. SHAP analysis highlighted MFCC-derived features as key contributors to model decisions, providing transparent interpretability. Overall, the study presents preliminary evidence linking acoustic characteristics to depressive symptoms and outlines a reproducible analytical workflow, while underscoring the need for substantially larger and more diverse datasets to establish clinically meaningful predictive validity.

## 1. Introduction

Major Depressive Disorder (MDD) is one of the most prevalent mental health conditions worldwide, affecting approximately 280 million individuals and imposing substantial socioeconomic burdens, including impaired functioning, reduced quality of life, and increased suicide risk (World Health Organization, n.d.). The urgency for early detection has intensified since the COVID-19 pandemic, which led to a more than 25% increase in global depression and anxiety prevalence (World Health Organization, 2022). Traditional diagnostic assessments such as the Patient Health Questionnaire-9 (PHQ-9) are clinically validated and widely used, yet they rely on subjective self-report and remain limited by recall bias and accessibility constraints (Kroenke et al., 2001). These limitations have stimulated growing interest in speech-derived biomarkers as objective, non-invasive, and scalable indicators of mental health status (Cummins et al., 2015; Hönig et al., 2014; Dogrucu et al., 2020; I.-M. Chen et al., 2022). Human speech conveys rich nonverbal information reflecting affective, cognitive, and physiological states. Beyond clinical observation, speech has also been widely used in computational mental health and affective computing research (Schuller et al., 2009). Numerous studies have documented acoustic alterations in individuals with depression, including increased jitter and shimmer (Hönig et al., 2014), disrupted prosody (Dumpala et al., 2023), and systematic spectral differences between patients and healthy controls (Menne et al., 2024). Recent findings further indicate that automated speech analysis can detect subtle depressive markers even in non-clinical populations (König et al., 2022). Mobile and digital health research has further demonstrated the feasibility of remote monitoring, linking speech diaries, sentiment, and vocal behavior to depressive symptom severity (Ben-Zeev et al., 2015; Nickels et al., 2021). Recent work using the publicly available MODMA dataset has advanced speech-based depression screening, employing traditional machine-learning models and deep neural networks (X. Chen & Pan, 2021; Wasserzug et al., 2022; Gheorghe et al., 2023). While these studies achieved promising binary classification performance, they largely focused on diagnosis-based discrimination (MDD vs. HC) and provided limited examination of continuous, clinically validated severity scales such as the PHQ-9. Additionally, many existing approaches rely on complex “black-box” architectures that hinder interpretability, a major barrier to clinical adoption (Al Hanai et al., 2018; Dogrucu et al., 2020).

To address these gaps, the present study pursued two complementary objectives. First, we examined correlations and group-level associations between acoustic features and PHQ-9 scores to identify clinically grounded acoustic markers of depressive symptomatology (Objective 1). Second, we evaluated an exploratory binary screening task distinguishing individuals with MDD from healthy controls using the same acoustic feature set (Objective 2), incorporating three feature-selection strategies—Recursive Feature Elimination (RFE), Principal Component Analysis (PCA), and Random Forest–based selection—along with Shapley Additive Explanations (SHAP) for interpretability.

To strengthen the analytical execution of Objective 1 and more directly link acoustic markers to clinically meaningful symptom burden, we additionally performed a supplementary severity-based classification analysis using a PHQ-9 ≥ 10 threshold. This analysis tested whether the acoustic features most strongly correlated with PHQ-9 scores also contain predictive information for distinguishing moderate or greater depressive symptoms from mild or minimal symptoms. Given the exploratory nature and limited sample size of the MODMA dataset (*n* = 52), the present study did not attempt regression or fine-grained ordinal modeling of continuous PHQ-9 scores. Instead, PHQ-9 served as the basis for severity-focused correlation and group analyses (Objective 1), and the binary diagnostic label (MDD vs. HC) was reserved for the screening-oriented predictive modeling in Objective 2.

## 2. Methods

### 2.1. Participants and Dataset

This study employed the publicly available Multi-modal Open Dataset for Mental-disorder Analysis (MODMA), which was specifically designed for research on voice-based mental health prediction (Zhu et al., 2022). The dataset includes voice recordings and psychological assessment data from 52 adult participants recruited at the Second Hospital of Lanzhou University in China. Among these participants, 23 were clinically diagnosed with Major Depressive Disorder (MDD), while the remaining 29 individuals with no history of psychiatric illness served as healthy controls (HC).

Table 1 summarizes the demographic and clinical characteristics of the participants. No significant differences were observed between groups in age or gender distribution. However, the HC group had significantly higher years of education than the MDD group (*p* < 0.001). As expected, PHQ-9 scores were substantially higher in the MDD group compared to the HC group (*p* < 0.001).

Each participant provided speech samples under four standardized conditions, which consisted of a self-introduction, the reading of predefined words, the description of a picture, and a short segment of free speech. All recordings were collected in a controlled acoustic environment and stored in high-quality .wav format. Alongside the speech data, the dataset also contains demographic information such as age, gender, and education level, as well as responses to validated psychological assessments.

Although MODMA is publicly accessible, the use of human subject data in this research was reviewed and approved by the Institutional Review Board (IRB) of Dankook University (Approval No. DKU-2024-11-030-002), ensuring that the study adhered to ethical standards.

### 2.2. Psychological Scales and Target Definition

The Patient Health Questionnaire-9 (PHQ-9) was employed to evaluate the severity of depressive symptoms. The PHQ-9 is a widely validated and clinically accepted self-report instrument that is routinely used for both screening and monitoring depression (Kroenke et al., 2001). In this study, PHQ-9 scores served two complementary purposes. First, they provided a standardized and clinically reliable measure of depression severity, enabling the assessment of symptom burden across participants. Second, they were used as a reference for correlation analyses with acoustic features, thereby linking vocal markers to a validated psychological scale.

In line with the dual aims of the study, PHQ-9 scores served as the reference for symptom severity in correlation and group-level analyses, whereas the binary diagnosis label (MDD vs. HC) was used as the supervised target for exploratory screening models. Because of the small sample size and single-site design (*n* = 52), we did not attempt to construct a regression or ordinal model predicting PHQ-9 scores; such severity modeling is reserved for future work with larger and more heterogeneous datasets.

### 2.3. Acoustic Feature Extraction and Preprocessing

Acoustic features were extracted from the voice recordings using openSMILE (Open Speech and Music Interpretation by Large-space Extraction), a widely adopted toolkit for speech and paralinguistic analysis (Eyben et al., 2010). The emo_large.conf configuration, which has been validated in emotion and clinical voice research, was applied to comprehensively capture relevant parameters. The extracted feature set included Mel-Frequency Cepstral Coefficients (MFCCs), pitch, jitter, shimmer, and additional indicators such as formants (vocal resonance), harmonicity (voice quality), and log-energy (vocal intensity).

For each participant, the extracted features from multiple utterances were averaged into a single representative vector, thereby reducing intra-individual variability and ensuring consistency in acoustic characterization. Prior to analysis, samples containing missing values were excluded, and demographic variables were encoded (e.g., gender coded as 0 = male and 1 = female). All acoustic features were then standardized to a common scale to facilitate comparability across participants. The resulting processed dataset was subsequently used for correlation analysis with PHQ-9 scores as well as for training the predictive models.

### 2.4. Correlation and Severity-Based Analyses

To examine the relationships between acoustic features and depressive symptom severity (Objective 1), we first conducted Spearman’s rank correlation analysis between all 6553 extracted acoustic features and PHQ-9 scores. Spearman’s ρ was selected as a robust non-parametric measure capable of capturing monotonic and non-linear associations. Features with an absolute correlation of |ρ| ≥ 0.3 were considered to reflect at least moderate associations. To reduce false positives due to multiple comparisons, *p*-values were adjusted using the Benjamini–Hochberg False Discovery Rate (FDR) procedure. Based on this analysis, the five acoustic features showing the strongest correlations with PHQ-9 scores were selected for subsequent interpretation. In addition, independent two-sample t-tests and Cohen’s d effect sizes were computed to assess corresponding group-level differences between MDD and HC participants.

To more directly connect acoustic markers with clinically defined symptom burden and to complement the continuous correlation analysis, we also performed a supplementary severity-based classification using a PHQ-9 threshold of 10 (indicative of moderate-or-greater depressive symptoms). For this analysis, we trained a logistic regression classifier with standardization (StandardScaler + Logistic Regression) using only the five MFCC-derived features that demonstrated the strongest correlations with PHQ-9 scores. Model performance was evaluated with stratified 5-fold cross-validation repeated three times (15 folds total), yielding cross-validated estimates of AUC (primary metric), along with accuracy, precision, and recall. This severity-based analysis provides an additional perspective on whether acoustic features associated with continuous PHQ-9 severity also exhibit discriminative value when PHQ-9 scores are coarsely categorized, thereby strengthening the analytical execution of Objective 1. Detailed numerical results for this supplementary analysis are reported in the Results section.

### 2.5. Model Development and Evaluation

To construct predictive models for depression classification, four machine-learning pipelines were developed, each incorporating a distinct feature-selection or dimensionality-reduction strategy. The first pipeline combined Recursive Feature Elimination (RFE) with Extreme Gradient Boosting (XGBoost). RFE iteratively removed less informative features, while XGBoost—a gradient boosting method effective for structured high-dimensional data—captured potentially non-linear relationships. The second pipeline applied Principal Component Analysis (PCA) followed by XGBoost classification. PCA was selected to reduce the 6553-dimensional feature space into orthogonal components, thereby mitigating multicollinearity, suppressing high-variance noise, and decreasing the risk of overfitting. The third pipeline employed Random Forest (RF) to estimate feature importance and used the top-ranked features to train an XGBoost classifier, leveraging the ensemble model’s robustness to feature redundancy.

For comparison, a baseline Logistic Regression model was constructed using all 6553 acoustic features without feature selection. Logistic Regression, with strong L2 regularization and a linear decision boundary, served as a stability-focused benchmark in the small-sample, high-dimensional setting. All models were trained and validated using stratified 5-fold cross-validation repeated three times to reduce variance associated with random data partitions. Prior to training, all features were standardized using StandardScaler. Because class imbalance can exacerbate variance and bias in small datasets, the Synthetic Minority Oversampling Technique (SMOTE) was applied conservatively, with oversampling ratios restricted to avoid generating unrealistic synthetic patterns while still mitigating extreme imbalance.

Model hyperparameters were optimized using grid search with cross-validation, and final performance was evaluated on an independent test set. Evaluation metrics included the Area Under the Receiver Operating Characteristic Curve (AUC-ROC) as the primary metric, along with accuracy, precision, recall, F1 score, and confusion matrices. This multi-metric evaluation provided a comprehensive assessment of predictive behavior under conditions prone to overfitting. The supervised task in this study was MDD versus HC classification, representing an exploratory screening scenario rather than direct severity prediction. This design choice reflects both the study’s exploratory aims and the limited statistical power available for modeling PHQ-9 scores as a continuous or ordinal variable.

### 2.6. Model Interpretation and Feature Contribution Analysis

To enhance transparency of the binary classification framework, Shapley Additive Explanations (SHAP) were applied to the best-performing model, the PCA + XGBoost pipeline. SHAP is a model-agnostic interpretability method grounded in cooperative game theory that quantifies the marginal contribution of each feature to the probability of predicting Major Depressive Disorder (MDD = 1) versus healthy control (HC = 0). Importantly, SHAP does not estimate or model continuous depression severity (e.g., PHQ-9 scores); rather, it explains how individual acoustic features influence the model’s binary decision boundary.

At the global level, SHAP beeswarm plots were generated to visualize the overall distribution, direction, and magnitude of feature contributions across participants. MFCC-derived spectral features—particularly those from the sixth coefficient—exerted the strongest influence on increasing the model’s predicted probability of MDD. At the local level, SHAP force plots illustrated how specific acoustic feature values supported or opposed an MDD prediction for an individual participant, thereby providing case-level transparency into the model’s decision process. Although SHAP and PHQ-9 correlation analyses address distinct analytic aims—binary discrimination (Objective 2) versus severity association (Objective 1)—several MFCC-based features appeared prominently in both analyses. This overlap should not be interpreted as evidence that the classifier models PHQ-9 severity directly. Instead, it reflects a structural property of the dataset: PHQ-9 scores sharply differentiate the MDD and HC groups, and therefore acoustic features encoding PHQ-9-linked severity differences naturally become influential for the binary classifier. In this sense, the overlap provides preliminary clinical face validity, indicating that the classifier relies on plausible and severity-aligned acoustic cues—rather than spurious noise—when distinguishing MDD from HC.

Overall, the SHAP analysis demonstrates that, despite modest predictive performance, the PCA + XGBoost classifier bases its decisions on psychologically and physiologically interpretable vocal characteristics. This supports the transparency and clinical coherence of the modeling framework and offers a foundation for future development of responsible, interpretable speech-based screening tools.

## 3. Results

### 3.1. Associations with PHQ-9 Severity

Spearman’s rank correlation analysis identified several acoustic features—particularly those derived from the sixth Mel-Frequency Cepstral Coefficient (MFCC)—as strongly associated with PHQ-9 depression severity. Among the 6553 extracted features, mfcc_sma[6]_linregc2 (ρ = 0.56, FDR = 0.001), mfcc_sma[6]_amean (ρ = 0.55, FDR = 0.001), and mfcc_sma[6]_quartile2 (ρ = 0.55, FDR = 0.001) exhibited the most robust correlations with PHQ-9 scores. Independent two-sample t-tests further revealed significant group-level differences between MDD and HC participants on these MFCC-related features, with large effect sizes (e.g., mfcc_sma[6]_amean: t = −4.16, *p* < 0.001, Cohen’s d = −1.18). These findings suggest that higher depressive symptom severity is systematically reflected in increased spectral variability and reduced vocal stability.

Taken together, the correlation and group-difference analyses consistently highlight MFCC-derived spectral features—especially those reflecting spectral slope and average energy distribution—as meaningful acoustic markers of PHQ-9 severity. These results directly address Objective 1 by demonstrating systematic and clinically interpretable associations between acoustic patterns and validated depression severity scores. Table 2 summarizes the top five PHQ-9–correlated acoustic features.

To further strengthen Objective 1 and evaluate whether severity-linked acoustic markers also support coarse symptom categorization, an additional severity-based classification analysis was conducted using a PHQ-9 threshold of 10 (moderate-or-greater symptoms). A logistic regression classifier trained on the five PHQ-9–correlated MFCC features achieved a cross-validated AUC of 0.78 (SD = 0.15), with an accuracy of 0.73 (SD = 0.16), precision of 0.76 (SD = 0.23), and recall of 0.61 (SD = 0.20) across 15 folds. These findings indicate that the same acoustic features associated with continuous PHQ-9 severity also provide discriminative value for distinguishing moderate-or-greater symptom burden from mild or minimal symptoms, reinforcing their clinical relevance. Table 3 summarizes the severity-based classification results.

### 3.2. Feature Selection Results

Across all three feature-selection pipelines—RFE + XGBoost, PCA + XGBoost, and RF + XGBoost—MFCC-derived acoustic features consistently emerged as the most informative predictors for distinguishing individuals with MDD from healthy controls. In particular, variables such as mfcc_sma[6]_linregc2 and mfcc_sma[6]_amean were repeatedly ranked among the highest contributors to the binary classification task. These features also appeared among the strongest correlates of PHQ-9 severity in Section 3.1, reflecting a degree of overlap between severity-related acoustic patterns and those used in the diagnostic classification model. However, these overlaps do not indicate that the classifier models PHQ-9 severity; rather, they reflect methodological consistency across distinct analytic goals—severity association (Objective 1) and diagnostic discrimination (Objective 2).

Each feature-selection method contributed complementary strengths. Recursive Feature Elimination (RFE) iteratively removed less informative predictors, retaining features with the greatest utility for the MDD-versus-HC decision boundary. Principal Component Analysis (PCA) reduced the 6553-dimensional feature space into uncorrelated components capturing more than 85% of the total variance; several high-loading components were driven by MFCC-based spectral characteristics indicative of vocal instability. Random Forest–based selection further reinforced these findings by assigning high importance scores to MFCC-derived measures within an ensemble-driven framework that is robust to noise and feature redundancy.

The convergence of results across these independent selection strategies underscores the robustness of MFCC-based spectral characteristics—particularly those capturing spectral slope, variability, and average energy distribution—as discriminative cues for the binary diagnostic task. Importantly, these features were selected to optimize binary MDD-versus-HC classification performance (Objective 2), not to model continuous PHQ-9 severity. Nevertheless, their overlap with severity-correlated features identified in Section 3.1 enhances the interpretability and potential clinical relevance of the predictive framework without conflating diagnostic discrimination with severity estimation.

### 3.3. Model Performance and Comparative Evaluation

**Figure 1:**
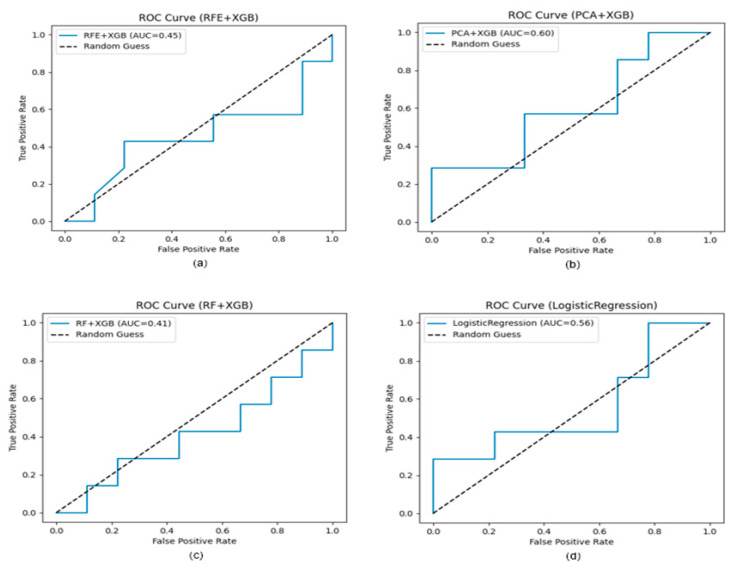
ROC Curves of Predictive Models: (**a**) RFE + XGBoost (**b**) PCA + XGBoost (**c**) RF + XGBoost (**d**) Logistic Regression.

In line with Objective 2, which aimed to explore the feasibility of voice-based binary screening of Major Depressive Disorder (MDD), four predictive pipelines—RFE + XGBoost, PCA + XGBoost, RF + XGBoost, and Logistic Regression—were evaluated using repeated stratified cross-validation and an independent test set. The results are summarized in Table 4, and the ROC curves for each model are presented in Figure 1.

During cross-validation, both RFE + XGBoost and RF + XGBoost achieved relatively high AUC values (0.76 each), suggesting that these pipelines captured complex feature interactions within the training data. However, their test-set AUCs dropped sharply (0.45 and 0.41), indicating substantial overfitting. This pattern likely reflects structural properties of the dataset: an extremely high-dimensional acoustic feature space (6553 features) combined with a limited number of participants (*n* = 52) increases the risk that recursive elimination procedures or tree-based importance measures identify dataset-specific variance rather than generalizable acoustic markers. Such methods tend to overweight high-variance features and are particularly vulnerable to instability under small-sample, high-dimensional conditions. Logistic Regression, which used all acoustic features without feature selection, achieved modest but more stable performance (test AUC = 0.56). Although its linear decision boundary limits its capacity to model complex interactions, its relative stability underscores the importance of model simplicity and stronger regularization when sample size is constrained.

Among all evaluated models, PCA + XGBoost achieved the most balanced performance, with a cross-validated AUC of 0.75 and the highest test-set AUC (0.60). PCA’s dimensionality reduction mitigated overfitting by transforming correlated acoustic features into orthogonal components, reducing redundancy and compressing the feature space while preserving informative variance. This structural advantage likely enabled the model to generalize more effectively to unseen data. Although the performance gains over Logistic Regression were incremental, PCA + XGBoost demonstrated the strongest generalization across pipelines, highlighting the importance of variance-preserving dimensionality reduction in limited-sample contexts.

Taken together, these findings illustrate a clear trade-off between model complexity and generalization. High-capacity pipelines (RFE- and RF-based) performed well on training folds but failed to generalize due to variance inflation and limited sample support, whereas PCA-based dimensionality reduction yielded more robust test performance. Based on this balance of predictive depth and generalization, the PCA + XGBoost pipeline was selected for subsequent SHAP-based interpretability analyses (Section 3.4). Future work incorporating larger sample sizes, stronger regularization, or representation learning techniques may further mitigate overfitting and improve the reliability of voice-based depression screening models.

### 3.4. Model Interpretation with SHAP

To improve interpretability of the binary classification framework, Shapley Additive Explanations (SHAP) were applied to the best-performing pipeline (Lundberg & Lee, 2017), PCA + XGBoost. SHAP quantifies the marginal contribution of each acoustic feature to the probability of predicting Major Depressive Disorder (MDD = 1) versus healthy control (HC = 0), providing both global and local explanations of the model’s decision process.

**Figure 2:**
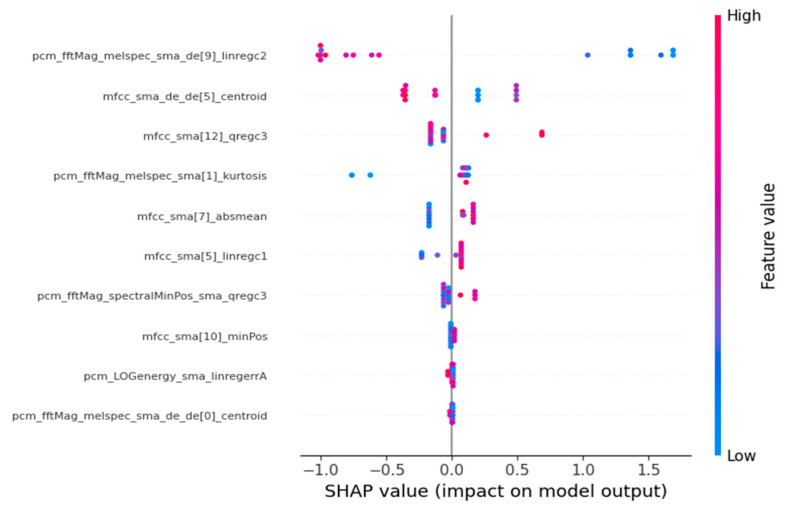
SHAP Beeswarm Plot for Feature Importance in PCA + XGBoost Model.

At the global level, SHAP beeswarm plots (Figure 2) showed that MFCC-derived features—particularly those from the sixth coefficient (e.g., mfcc_sma[6]_linregc2, mfcc_sma[6]_amean)—exerted the strongest influence on increasing the model’s predicted probability of MDD. Higher or more unstable spectral values pushed the model output toward the MDD class, whereas more stable patterns supported HC predictions. Importantly, SHAP reflects contributions to the binary discrimination task and does not directly estimate PHQ-9 severity.

At the local level, SHAP force plots demonstrated how participant-specific acoustic values shaped classification outcomes. For example, increased MFCC slope or greater spectral variability contributed positively to MDD predictions, whereas lower variability shifted predictions toward HC. These individualized explanations provide transparent insight into how the classifier arrives at each decision.

It is analytically important to distinguish the roles of SHAP and the PHQ-9 correlation analysis: the latter examines associations with continuous depression severity (Objective 1), whereas SHAP interprets binary diagnostic decisions (Objective 2). Nevertheless, the fact that several MFCC-related features appear prominently in both analyses is not merely methodological coincidence. Rather, it reflects a structural property of the dataset: PHQ-9 scores strongly differentiate MDD from HC in this sample, and therefore acoustic features that encode PHQ-9-linked severity differences naturally become influential for the binary classifier as well. This alignment strengthens the clinical face validity of the model, indicating that its decision-making relies on acoustically measurable markers that correspond to clinically meaningful differences in depressive symptom burden.

Overall, while predictive performance remains modest, the SHAP analysis shows that the classifier depends on psychologically and physiologically plausible vocal cues—particularly severity-linked spectral characteristics—providing a transparent and clinically interpretable basis for future development of responsible voice-based screening models.

## 4. Discussion

This study examined associations between voice-derived acoustic features and PHQ-9 depression severity, and explored the feasibility of predicting Major Depressive Disorder (MDD) in an exploratory binary screening task. Among the predictive pipelines, the PCA + XGBoost model achieved the most reliable generalization, while SHAP analysis provided interpretable insights into how specific acoustic features contributed to classification. Based on these findings, the discussion highlights the significance of feature selection, the comparative performance of predictive models, their interpretability, and the clinical implications of this approach.

### 4.1. Significance of Feature Selection Techniques

Feature selection played a central role in clarifying which acoustic characteristics were most meaningfully associated with depressive symptoms and in improving the interpretability of the predictive framework. Consistent with Objective 1, the initial correlation analysis identified several MFCC-derived spectral features—particularly those reflecting spectral slope and average energy—as moderately associated with PHQ-9 severity, echoing prior evidence that depressive states manifest through reduced vocal stability and altered prosodic patterns (Hönig et al., 2014; Dumpala et al., 2023; Menne et al., 2024).

Building on these severity-focused findings, three complementary feature selection techniques—Recursive Feature Elimination (RFE), Principal Component Analysis (PCA), and Random Forest–based importance ranking—were applied to identify features informative for the exploratory binary screening task (Objective 2). Despite methodological differences, all approaches consistently highlighted MFCC-related dimensions, suggesting that these spectral patterns contain stable information relevant for distinguishing MDD from healthy controls.

The convergence between (a) PHQ-9 severity correlations and (b) feature importance across multiple selection pipelines provides a coherent picture of MFCC-derived measures as key acoustic markers. Crucially, these analyses address distinct goals: correlations identify severity-linked acoustic characteristics (Objective 1), while feature selection identifies features relevant for binary discrimination (Objective 2). Their overlap should therefore be interpreted as methodological consistency rather than evidence that the classifier directly models PHQ-9 severity.

In addition to the correlation and group-difference analyses, we conducted a supplementary severity-based classification using a PHQ-9 ≥ 10 threshold to strengthen the analytical execution of Objective 1. The logistic regression model trained on the five MFCC-derived features most strongly associated with PHQ-9 demonstrated moderate discriminative ability (AUC = 0.78), indicating that these severity-linked acoustic markers are not only statistically correlated with PHQ-9 scores but also informative for distinguishing clinically meaningful levels of symptom burden. This supplementary analysis provides convergent evidence supporting the relevance of the identified acoustic markers to depressive symptom severity.

Finally, although MFCC patterns were stable within the present sample, recent cross-linguistic studies indicate that feature robustness may vary across languages and recording contexts. Future research should therefore aim to identify feature subsets that generalize across diverse populations, a challenge highlighted in emerging cross-corpus and cross-lingual depression detection research (Qin et al., 2025; Cummins et al., 2023).

### 4.2. Comparative Performance of Predictive Models

The comparative evaluation of predictive models highlighted the substantial challenges of achieving generalizable performance when modeling high-dimensional acoustic data under severe sample-size constraints. Although RFE + XGBoost and RF + XGBoost achieved relatively high cross-validated AUC values (0.76 each), their test-set AUCs fell below chance level (0.45 and 0.41), indicating severe overfitting. This overfitting is not an algorithm-specific artifact but a structural consequence of the data configuration. With 6553 acoustic predictors and only 52 participants, the feature-to-sample ratio is extremely imbalanced, creating an ill-posed learning problem in which minor idiosyncrasies of the training data are easily mistaken for meaningful signal. Methods such as Recursive Feature Elimination and Random Forest–based importance ranking tend to amplify high-variance, sample-specific fluctuations when sample sizes are small. RFE iteratively removes features based on unstable marginal importance estimates, while tree-based feature importance is known to vary substantially when the number of observations is insufficient to stabilize split-selection statistics. As a result, both pipelines captured training-set structure but failed to generalize, consistent with prior reports documenting instability of feature-selection models in small clinical datasets.

Logistic Regression, despite its limited representational capacity, produced more stable performance (test AUC = 0.56). Its comparative robustness illustrates that strong regularization and linear decision boundaries can mitigate—but not eliminate—the risks of variance inflation when dimensionality far exceeds sample size. Among all pipelines, PCA + XGBoost yielded the most balanced results (test AUC = 0.60). PCA reduced the 6553-dimensional feature space into a set of orthogonal components that captured the dominant variance structure while filtering out high-frequency noise and collinearity. This bottleneck transformation effectively reduced the intrinsic dimensionality of the problem, enabling the downstream classifier to rely on stable, variance-preserving components rather than unstable raw features. The incremental yet consistent improvement of PCA + XGBoost suggests that dimensionality reduction is particularly critical when modeling clinical acoustic datasets with severe feature-to-sample imbalances.

These generalization challenges mirror a broader pattern observed in cross-cohort and cross-corpus speech depression research, in which models trained on small datasets frequently degrade when applied to new populations or recording environments (Qin et al., 2025; Cummins et al., 2023). Together, the results reinforce that, within the constraints of the present dataset, the predictive signal available from acoustic features is weak and easily overshadowed by noise. The relative stability of PCA + XGBoost should therefore be interpreted not as evidence of clinical feasibility, but as a methodological indicator that variance-preserving dimensionality reduction is essential for early-stage exploratory modeling. Clinically meaningful predictive validity will require substantially larger, multilingual, and heterogeneous datasets to stabilize feature learning and reduce susceptibility to overfitting.

### 4.3. Interpretability and Clinical Implications

A transparent and clinically interpretable prediction process is essential in mental health applications, where clinicians must understand how a model arrives at its decisions. To improve interpretability, SHAP analysis was applied to the best-performing pipeline (PCA + XGBoost). Importantly, SHAP describes the contribution of each acoustic feature to the binary prediction outcome—whether a participant is classified as MDD (1) or HC (0). SHAP does not estimate or model continuous PHQ-9 severity. At the global level, SHAP beeswarm plots indicated that MFCC-related features—especially those derived from the sixth coefficient—had the strongest influence on the model’s probability of predicting MDD. Higher or more variable values in these features shifted predictions toward the MDD class, whereas more stable spectral patterns favored HC predictions. At the local level, SHAP force plots illustrated how individual acoustic feature values supported or opposed an MDD prediction, providing case-level transparency into the model’s decision process.

Although the correlation analysis (Objective 1) and the SHAP analysis (Objective 2) address conceptually distinct questions—severity association versus binary discrimination—the appearance of the same MFCC-based features in both analyses is not a methodological coincidence. The PHQ-9 scores in this dataset sharply differentiate the MDD and HC groups, and therefore acoustic features that encode PHQ-9-linked severity differences naturally become influential for the binary classifier as well. This structural relationship reflects how depression severity aligns with diagnostic group differences in the MODMA dataset. Accordingly, the overlap between severity-correlated features and SHAP-ranked features should be interpreted as evidence of clinical face validity: the classifier’s decision-making relies on the same acoustic characteristics that track clinically meaningful differences in symptom burden. Rather than suggesting that the classifier models PHQ-9 directly, the SHAP findings demonstrate that the model depends on plausible severity-linked vocal cues—such as spectral slope and stability—that are consistent with established clinical voice research.

The clinical implications of these results remain preliminary. While the classifier’s reliance on severity-linked acoustic features enhances interpretability and clinical coherence, overall predictive performance was modest and insufficient for deployment. Interpretable machine-learning approaches may, however, provide value as adjunctive tools when combined with multimodal data or embedded within broader assessment pipelines. Consistent with recent discussions in clinical speech AI (Berisha & Liss, 2024), transparent and severity-aligned decision-making represents an important foundation for future development, even though substantial improvements in predictive accuracy and external validation are necessary before real-world application.

### 4.4. Limitations and Future Directions

This study has several important limitations that should be acknowledged to properly contextualize the findings. First, the relatively small sample size (*n* = 52) and single-site data collection substantially limit statistical power and external validity. These constraints are particularly problematic for high-dimensional acoustic modeling, where 6553 features vastly exceed the number of observations, producing an extreme feature-to-sample imbalance. Such configurations are structurally ill-posed: even minor idiosyncrasies of the training data can be mistaken for meaningful signal, making severe overfitting statistically inevitable. This aligns with cross-cohort findings showing degradation of speech-based depression models when applied across heterogeneous populations or acoustic environments (Di et al., 2021; Qin et al., 2025; Gonzalez-Machorro et al., 2025).

Second, the exclusive reliance on acoustic features limits the scope of depressive symptom characterization. Depression is a multimodal condition expressed not only through vocal acoustics but also linguistic content, facial movements, behavioral patterns, wearable sensor signals, and contextual factors. Future research should therefore integrate multimodal information—such as speech transcripts, facial expression analysis, mobile sensing, ecological momentary assessment, or clinical metadata—to construct richer and more ecologically valid digital phenotypes.

Third, although SHAP analysis improves interpretability, the identified relationships remain correlational. SHAP explains how acoustic features influence binary classification decisions in the PCA + XGBoost model but does not provide causal evidence linking these features to PHQ-9 severity or underlying psychopathology. Establishing mechanistic associations will require longitudinal study designs that track dynamic symptom changes across illness trajectories, treatment response, or relapse patterns.

Fourth, and most critically, the predictive performance of the binary classification models was modest (best test AUC = 0.60), reflecting the structural challenges of small-sample, high-dimensional modeling. Methods such as RFE and Random Forest–based feature importance are known to produce unstable feature rankings under limited sample sizes, amplifying high-variance, dataset-specific noise. Although PCA partially mitigated these issues by reducing dimensionality and removing collinearity, the remaining predictive signal remains weak. Addressing these structural causes of overfitting will require substantially larger datasets as well as modern representation-learning strategies—such as transfer learning, pre-trained speech embeddings (e.g., wav2vec 2.0), foundation speech models, self-supervised learning, or domain adaptation techniques—that are specifically designed to overcome distributional shifts and variance inflation in cross-corpus speech modeling (Gómez-Zaragozá et al., 2025; Baevski et al., 2020).

Lastly, because of the limited statistical power, the study did not attempt regression or ordinal modeling of PHQ-9 severity. Reliable modeling of continuous symptom distributions remains an important goal for future work but will require substantially larger and more diverse datasets.

In summary, this study provides an initial foundation for understanding how acoustic features relate to depressive symptoms and for exploring their potential in preliminary screening models. Future investigations using larger samples, multimodal data, rigorous external validation, and longitudinal designs will be essential for advancing acoustic markers toward clinically meaningful and generalizable digital biomarkers. These priorities align with recent systematic reviews emphasizing that data diversity and robust cross-cohort validation are prerequisites for reliable speech-based depression detection systems (Mao et al., 2023).

## 5. Conclusions

This exploratory study examined whether voice-derived acoustic features reflect depressive symptom severity and contain preliminary signals for distinguishing Major Depressive Disorder (MDD) from healthy controls (HC). MFCC-derived spectral features were consistently associated with PHQ-9 scores and showed discriminative value in a supplementary severity-based classification using a PHQ-9 ≥ 10 threshold, strengthening the analytical execution of Objective 1. Notably, the severity-linked MFCC features identified in correlation analyses were also among the most influential predictors in the binary classification model, as revealed by SHAP. This alignment between severity-related acoustic cues and model decision-making offers preliminary clinical face validity, even though the overall predictive performance remains insufficient for screening applications. Among the predictive pipelines, PCA + XGBoost achieved the most stable generalization, underscoring the methodological value of dimensionality reduction in small, high-dimensional datasets. Nevertheless, reliable predictive capability was not achieved, emphasizing the need for substantially larger, multilingual, and multi-site datasets—and for multimodal and longitudinal designs—to determine whether voice-derived acoustic markers can contribute meaningfully to clinically robust depression detection or monitoring systems.

## Figures and Tables

**Table 1:** Demographic and Clinical Characteristics of Participants.

Variable	MDD (*n* = 23)	HC (*n* = 29)	*p*-Value
Gender, *n* (%)			
Male	16 (69.6%)	20 (69.0%)	1.00
Female	7 (30.4%)	9 (31.0%)	
Age (years)	30.91 ± 9.74	31.55 ± 8.93	0.81
Education (years)	12.87 ± 3.75	16.76 ± 2.63	<0.001
PHQ-9	18.09 ± 4.44	2.52 ± 2.11	<0.001

**Table 2:** Top 5 acoustic features most strongly correlated with PHQ-9 severity.

Feature	Description	Spearman’s Rho	*p*-Value (Spearman)	FDR	*t*-Test	*p*-Value (*t*-Test)	Cohen’s d
mfcc_sma[6]_linregc2	Slope of MFCC (6th Coefficient)	0.56	*p* < 0.001	0.001	−3.93	*p* < 0.001	−1.11
mfcc_sma[6]_amean	Mean of MFCC (6th Coefficient)	0.55	*p* < 0.001	0.001	−4.16	*p* < 0.001	−1.18
mfcc_sma[6]_quartile2	Second Quartile of MFCC (6th Coefficient)	0.55	*p* < 0.001	0.001	−4.09	*p* < 0.001	−1.16
mfcc_sma[6]_peakMean	Peak Mean of MFCC (6th Coefficient)	0.54	*p* < 0.001	0.001	−3.93	*p* < 0.001	−1.11
mfcc_sma[6]_quartile1	First Quartile of MFCC (6th Coefficient)	0.53	*p* < 0.001	0.001	−4.02	*p* < 0.001	−1.13

**Table 3:** Severity-based classification performance using PHQ-9 ≥ 10 (Logistic Regression with the top 5 correlated MFCC features).

Metric	Mean	SD	Notes
AUC	0.78	0.15	15-fold CV
Accuracy	0.73	0.16	15-fold CV
Precision	0.76	0.23	Positive = PHQ-9 ≥ 10
Recall	0.61	0.20	Positive = PHQ-9 ≥ 10

**Table 4:** Performance Metrics of the Evaluated Predictive Models.

Model	CV Best AUC	Test AUC	Accuracy	Precision	Recall	F1 Score	Confusion Matrix
RFE + XGBoost	0.76	0.45	0.50	0.44	0.57	0.50	[[4, 5], [3, 4]]
PCA + XGBoost	0.75	0.60	0.56	0.50	0.57	0.53	[[5, 4], [3, 4]]
RF + XGBoost	0.76	0.41	0.44	0.40	0.57	0.47	[[3, 6], [3, 4]]
Logistic Regression	0.74	0.56	0.44	0.38	0.43	0.40	[[4, 5], [4, 3]]

## Data Availability

MODMA-DATASET. Available online: https://www.kaggle.com/datasets/mimino12/modma-dataset/data (accessed on 12 March 2025).
